# Supplementary material for: Food Security Interventions among Refugees around the Globe: A Scoping Review
Source: Nutrients. 2022 Jan 25;14(3):522. doi: 10.3390/nu14030522 (PMC8839314; doi:10.3390/nu14030522)
Supplement: Supplementary file 1 [file nutrients-14-00522-s001.zip › nutrients-1422423 - supplementary material Table S1.pdf]

Table S1. Sample search strategy from Ovid Medline.

No.	Sample search strategy
1.	Refugees/
2.	refuge*.mp. [mp=title, abstract, original title, name of substance word, subject heading word, floating sub- heading word, keyword heading word, organism supplementary concept word, protocol supplementary concept word, rare disease supplementary concept word, unique identifier, synonyms]
3.	asylum*.mp. [mp=title, abstract, original title, name of substance word, subject heading word, floating sub- heading word, keyword heading word, organism supplementary concept word, protocol supplementary concept word, rare disease supplementary concept word, unique identifier, synonyms]
4.	(Displaced adj2 (person? or people)).mp. [mp=title, abstract, original title, name of substance word, subject heading word, floating sub-heading word, keyword heading word, organism supplementary concept word, protocol supplementary concept word, rare disease supplementary concept word, unique identifier, synonyms]
5.	1 or 2 or 3 or 4
6.	food supply/ or famine/
7.	Food/ or "diet, food, and nutrition"/
8.	diet/ or diet, diabetic/ or diet, healthy/ or eating/ or drinking/ or feeding behavior/ or breast feeding/
9.	Malnutrition/
10.	famine*.mp. [mp=title, abstract, original title, name of substance word, subject heading word, floating sub- heading word, keyword heading word, organism supplementary concept word, protocol supplementary concept word, rare disease supplementary concept word, unique identifier, synonyms]
11.	(hunger or hungry).mp. [mp=title, abstract, original title, name of substance word, subject heading word, floating sub-heading word, keyword heading word, organism supplementary concept word, protocol supplementary concept word, rare disease supplementary concept word, unique identifier, synonyms]
12.	starv*.mp. [mp=title, abstract, original title, name of substance word, subject heading word, floating sub-heading word, keyword heading word, organism supplementary concept word, protocol supplementary concept word, rare disease supplementary concept word, unique identifier, synonyms]
13.	malnutrition.mp. [mp=title, abstract, original title, name of substance word, subject heading word, floating sub-heading word, keyword heading word, organism supplementary concept word, protocol supplementary concept word, rare disease supplementary concept word, unique identifier, synonyms]
14.	malnourish*.mp. [mp=title, abstract, original title, name of substance word, subject heading word, floating sub-heading word, keyword heading word, organism supplementary concept word, protocol supplementary concept word, rare disease supplementary concept word, unique identifier, synonyms]
15.	food*.mp. [mp=title, abstract, original title, name of substance word, subject heading word, floating sub-heading word, keyword heading word, organism supplementary concept word, protocol supplementary concept word, rare disease supplementary concept word, unique identifier, synonyms]
16.	(diet* or nutritio* or eat* or drink* or feed*).mp. [mp=title, abstract, original title, name of substance word, subject heading word, floating sub-heading word, keyword heading word, organism supplementary concept word, protocol supplementary concept word, rare disease supplementary concept word, unique identifier, synonyms]
17.	or/6-16
18.	5 and 17
